# Cellular cholesterol licenses *Legionella pneumophila* intracellular replication in macrophages

**DOI:** 10.15698/mic2023.01.789

**Published:** 2022-12-06

**Authors:** Edna Ondari, Ashley Wilkins, Brian Latimer, Ana-Maria Dragoi, Stanimir S. Ivanov

**Affiliations:** 1 Department of Microbiology and Immunology, Louisiana State University Health - Shreveport, Shreveport, LA 71130.; 2 Department of Molecular and Cellular Physiology, Louisiana State University Health - Shreveport, Shreveport, LA 71130.; 3 Innovative North Louisiana Experimental Therapeutics program (INLET), Feist-Weiller Cancer Center, Louisiana State University Health - Shreveport, Shreveport, LA 71130.

**Keywords:** Legionella pneumophila, macrophage, intracellular replication, cholesterol, niche homeostasis

## Abstract

Host membranes are inherently critical for niche homeostasis of vacuolar pathogens. Thus, intracellular bacteria frequently encode the capacity to regulate host lipogenesis as well as to modulate the lipid composition of host membranes. One membrane component that is often subverted by vacuolar bacteria is cholesterol - an abundant lipid that mammalian cells produce *de novo* at the endoplasmic reticulum (ER) or acquire exogenously from serum-derived lipoprotein carriers. *Legionella pneumophila* is an accidental human bacterial pathogen that infects and replicates within alveolar macrophages causing a severe atypical pneumonia known as Legionnaires' disease. From within a unique ER-derived vacuole *L. pneumophila* promotes host lipogenesis and experimental evidence indicates that cholesterol production might be one facet of this response. Here we investigated the link between cellular cholesterol and *L. pneumophila* intracellular replication and discovered that disruption of cholesterol biosynthesis or cholesterol trafficking lowered bacterial replication in infected cells. These growth defects were rescued by addition of exogenous cholesterol. Conversely, bacterial growth within cholesterol-leaden macrophages was enhanced. Importantly, the growth benefit of cholesterol was observed strictly in cellular infections and *L. pneumophila* growth kinetics in axenic cultures did not change in the presence of cholesterol. Microscopy analyses indicate that cholesterol regulates a step in *L. pneumophila* intracellular lifecycle that occurs after bacteria begin to replicate within an established intracellular niche. Collectively, we provide experimental evidence that cellular cholesterol promotes *L. pneumophila* replication within a membrane bound organelle in infected macrophages.

## INTRODUCTION

Replication within a unique membrane-bound organelle is a common feature in the lifecycle of many bacterial pathogens [1–4]. Often starting as a phagosome in the endocytic compartment, these bacteria-occupied vacuoles transition into organelles with novel molecular features via remodeling of host proteins and lipids through the highly coordinated actions of bacterial factors [3, 5]. Maturation of pathogen-occupied vacuoles into cellular organelles that support bacterial replication is frequently accomplished via rewiring of the host cell intracellular transport pathways for nutrient delivery [5–7]. Coordination of organelle expansion and pathogen replication is critical for niche homeostasis; therefore, host lipids and their biosynthesis pathways have emerged as critical regulators of intracellular replication for vacuolar pathogens both as organelle membrane building blocks as well as nutrients [8, 9].

The causative agent of Legionnaires' disease, *Legionella pneumophila* (Lp) is a prototypical intracellular bacterial pathogen that establishes and replicates within a unique endoplasmic reticulum (ER)-derived vacuole in amoebae and in macrophages [10–13]. The *Legionella*-containing vacuole (LCV) matures into an organelle that supports bacterial replication within four hrs of internalization by a process that involves recruitment and fusion with early secretory vesicles [14–16]. LCV maturation is orchestrated by the coordinated action of some of the over 300 Lp effector proteins translocated within the host cytosol by the Lp type IVb secretion system (T4bSS), known as the Dot/Icm apparatus [17, 18]. This translocation nanomachine is essential for intracellular survival and deletion mutants lacking single structural components of the Dot/Icm apparatus are avirulent because they fail to block endocytic maturation [19–21]. Dot/Icm effectors also regulate LCV integrity and expansion via distinct mechanisms. During maturation LCV membrane integrity depends on the rapid removal of the host endosomal regulator inositol 5-phosphatase OCRL1 (**O**culo**c**erebro**r**enal syndrome of **L**owe 1) by the Dot/Icm effector SdhA [22]. In macrophage infections, LCVs containing the *sdhA* deletion mutant rupture during maturation, release bacteria-derived molecules in the host cytosol and trigger pyroptosis – an inflammatory host cell death that restricts bacterial replication [23–25]. LCV instability in the absence of SdhA is alleviated by genetic deletion of a Lp encoded enzyme PlaA that contains phospholipase as well as cholesterol esterase activities and is secreted within the LCV lumen by the Lp Type II secretion system (T2SS) [23, 26–28]. Additionally, LCV membrane expansion is controlled in part by *de novo* lipogenesis [29, 30], triggered by the host master metabolic checkpoint serine/threonine kinase MTOR (**M**echanistic **t**arget **o**f **r**apamycin) that responds to cues from energy and nutrient sensing pathways [29, 31]. Lp activates MTOR signaling downstream of the amino-acid sensing pathway [29, 32] in infected macrophages by injecting Dot/Icm effectors which cause a selective blockade in host protein translation and thus increase the amount of free amino acids [32]. Disruption of *de novo* lipogenesis through MTOR inhibition can stifle LCV expansion causing membrane rupture, which can be overcome by supplementation with exogenous lipids [29]. Notably, cholesterol-rich LDLs (**L**ow-**d**ensity **l**ipoprotein) but not cholesterol-depleted serum rescue LCV expansion when MTOR is inhibited in macrophage infections indicating that cholesterol might play a role in LCV homeostasis [29]. Whether cellular cholesterol homeostasis impinges on Lp intracellular survival has not been directly investigated and thus, it is unclear whether cholesterol or another LDL packaged lipid species regulates LCV expansion.

In biological membranes cholesterol regulates packing and phase separation of phospholipids, thus directly impacting both membrane rigidity and permeability as well as the formation of lipid microdomains [33]. Cholesterol homeostasis is regulated at the ER membrane where cholesterol concentration is monitored, *de novo* production is carried out, and excess is packaged within lipid droplets [34]. The cholesterol in the ER membrane remains low at steady state because sterols are trafficked to peripheral organelles mainly via non-vesicular transport mechanisms resulting in a cholesterol concentration gradient within eukaryotic cells from low (ER) to high (plasma membrane and the endolysosomal network) [34–37]. Internalized serum-derived LDLs also supply cells with cholesterol and cholesteryl esters from which free cholesterol is extracted by lysosomal lipases, exported by the lysosomal proteins NPC1 and NPC2 (Niemann–Pick C) and disseminated throughout cellular membranes [34, 37]. Macrophages can accumulate and sequester excess cholesterol within lipid droplets following esterification by ACAT1 (**A**cyl coenzyme A:**c**holesterol **a**cyl**t**ransferase-1) or export cholesterol out of the cell [34, 38].

Bacterial pathogens exploit various aspects of cholesterol homeostasis (uptake, trafficking, production, storage and efflux) for different purposes including niche biogenesis and intracellular replication [39]. Vacuolar niches harboring *Anaplasma* [40, 41], *Chlamydia* [42], *Coxiella* [43] or *Erlichia* [44] accumulate cholesterol and all of these pathogens with the exception of *Coxiella* have been found to incorporate scavenged cholesterol into their cell envelope as well [42, 45]. Notably, genes encoding cholesterol modifying enzymatic domains have been identified in genomes of pathogenic and symbiotic bacteria [39, 46]. One example of anabolic reprograming is the Δ24 sterol reductase CUB1206 produced by *Coxiella burnetii* which has been demonstrated to complete the terminal step in ergosterol biosynthesis in eukaryotic cells [47]. Similarly, *Legionella drancourtii* encodes a putative eukaryotic-like Δ7 sterol reductase, which is highly expressed during amoeba co-cultures [46]. Conversely, Lp encodes three cholesterol acyltransferases – PlaA, PlaC and PlaD – which are secreted by the T2SS extracellularly or in the lumen of the LCV [26, 48, 49] and have been shown to esterify both cholesterol and ergosterol with short chain fatty acids *in vitro* [26]. Acylation generally precedes cholesterol sequestration within lipid droplets, indicating that Lp might have the capacity to modify cholesterol and thus alter the lipid composition of host membranes. Here, we investigate the role of cholesterol biosynthesis and trafficking in Lp intracellular replication within macrophage infections.

## RESULTS

### Generation of bioluminescent Lp strain for high-throughput, multivariable analysis of bacterial intracellular replication

**Figure 1:**
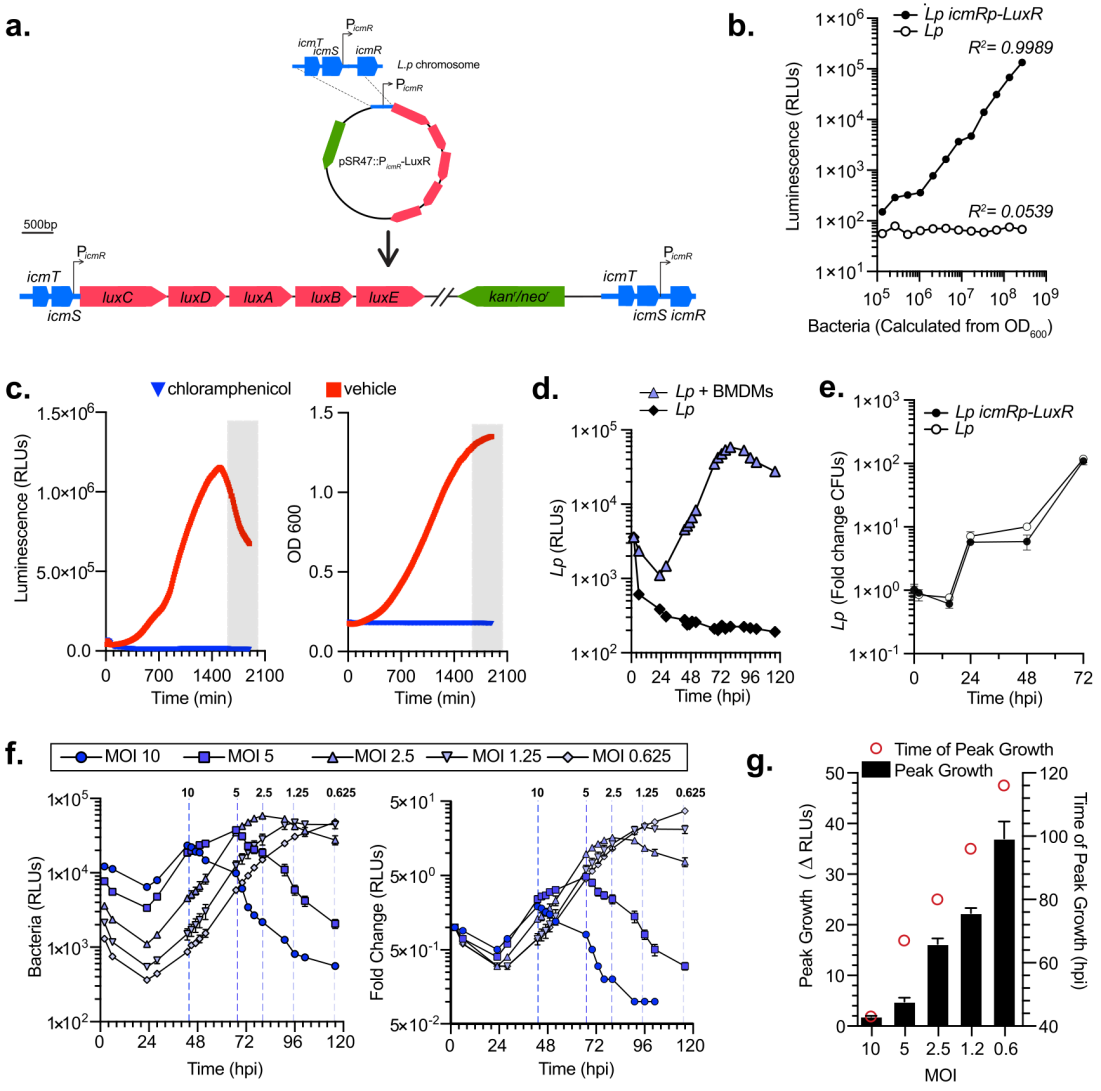
FIGURE 1: Construction and characterization of *L. pneumophila* strain encoding the LuxR operon. **(a)** Construction of the Lp *icmRp-LuxR* strain via single homologous recombination event with the suicide plasmid pSR47::*icmRp-LuxR*, which encodes the LuxR operon cloned downstream of a 863 nt region of homology containing the *icmR* promoter region from the Lp01 strain. **(b)** Luminescence output of the LuxR-encoding Lp as compared to the parental strain. Simultaneous luminescence and optical density measurements are shown from serial dilutions prepared from two-day heavy patches grown on CYA plates. **(c)** Luminescence output of the Lp *icmRp-LuxR* measured every ten min in axenically grown AYE liquid cultures in the presence/absence of chloramphenicol over the indicated time period. Each time point represents an average of a technical triplicate. **(d)** Bacterial growth of Lp *icmRp-LuxR* measured from bioluminescence output when bacteria were cultured in the absence or presence of BMDMs. **(e)** Growth comparison between in the indicated Lp strains in BMDMs infections measured by a CFU growth assay. **(f)** Growth kinetics of Lp *icmRp-LuxR* is shown for various MOIs. A vertical line indicates the peak of the growth curve for each infection. **(g)** The peak time and amplitude of the growth curve for each MOI from (f) are shown. **(d-g)** Each data point represents an average from technical triplicates ± SD. **(b-g)** Representative data from one of three biological replicates are shown.

Bioluminescence is an excellent reliable reporter for measuring bacterial growth kinetics in cellular infections, especially when robust, rigorous, multivariable analyses are needed [50, 51]. Collective light output from bioluminescent bacteria allows for direct measurement of bacterial cultures growth kinetics. Importantly, bioluminescence output is reflective of the metabolic state of the bacterium because the enzymology of light production requires ATP [52]. We engineered a bioluminescent *L. pneumophila* Lp01 strain (Lp *icmRp-LuxR*) by inserting the LuxR operon (*luxC luxD luxA luxB luxE*) from *Photorhabdus luminescens* on the Lp chromosome under the constitutive promoter of the Lp *icmR* gene using insertion-duplication mutagenesis (**Fig. 1a**). Light output by the bioluminescent Lp *icmRp-LuxR* strain allowed for quantitation of the bacteria population with a linear dynamic range of at least four orders of magnitude (**Fig. 1b**). In axenic growth culture, the bioluminescence signal produced by Lp *icmRp-LuxR* increased exponentially during log phase and peaked as bacteria entered stationary phase (**Fig. 1c**). In stationary phase, light output decreased sharply (**Fig. 1c**) consistent with the dependency of bioluminescence production on cellular metabolism [51]. As expected, the addition of the protein synthesis inhibitor chloramphenicol inhibited both bacterial replication and bioluminescence production (**Fig. 1c**). Thus, we conclude that bioluminescence output from the bacterial cultures containing Lp *icmRp-LuxR* adequately reflect the kinetics of bacterial growth in axenic cultures.

Next, we measured the growth of the Lp *icmR*p-*LuxR* strain in primary bone marrow-derived macrophages (BMDMs) infections for 120 hours where bioluminescence was measured at 18 discrete timepoints. After an initial decrease, bioluminescence increased exponentially peaking at 80 hours post infection (hpi) when bacteria were cultured with BMDMs (**Fig. 1d**). In contrast, bioluminescence production decreased to ∼5% of the light produced by the inoculum when Lp *icmR*p-*LuxR* was cultured in parallel without BMDMs, consistent with inability of the bacteria to replicate without host cells. Growth kinetics of Lp *icmR*p-*LuxR* in BMDMs infections was indistinguishable from the parental non-luminescent strain (**Fig. 1e**). Next, the ratio between Lp *icmR*p-*LuxR* and host cells was varied in the cell culture infections (**Fig. 1f-g**). The results showed that bioluminescence output from the infections peaked at an earlier timepoint when higher multiplicity of infection (MOI) was used and at later times when lower MOIs were used (**Fig. 1 f-g**). Because availability of host cells dictates maximum bacterial growth in these infection assays, the peak fold change in bacterial growth was highest when the lowest MOI was used which allowed for the most infection cycles to occur (**Fig. 1 f-g**). We conclude that kinetic measurements of light productions by Lp *icmR*p-*LuxR* during cellular infections adequately represent bacterial growth in a closed experimental system where BMDMs support intracellular bacterial replication but themselves do not divide due to absence of macrophage-specific growth factors, such as MCSF, which allows several sequential infection cycles to occur until macrophages are depleted. Thus, infections at the lower MOIs ultimately produced the highest amplitude in the growth curve (**Fig. 1g**) because the greatest number of infection cycles could occur before the exhaustion of viable host cells.

### Cholesterol and inhibitors of cholesterol biogenesis/trafficking do not alter Lp growth in axenic cultures

**Figure 2:**
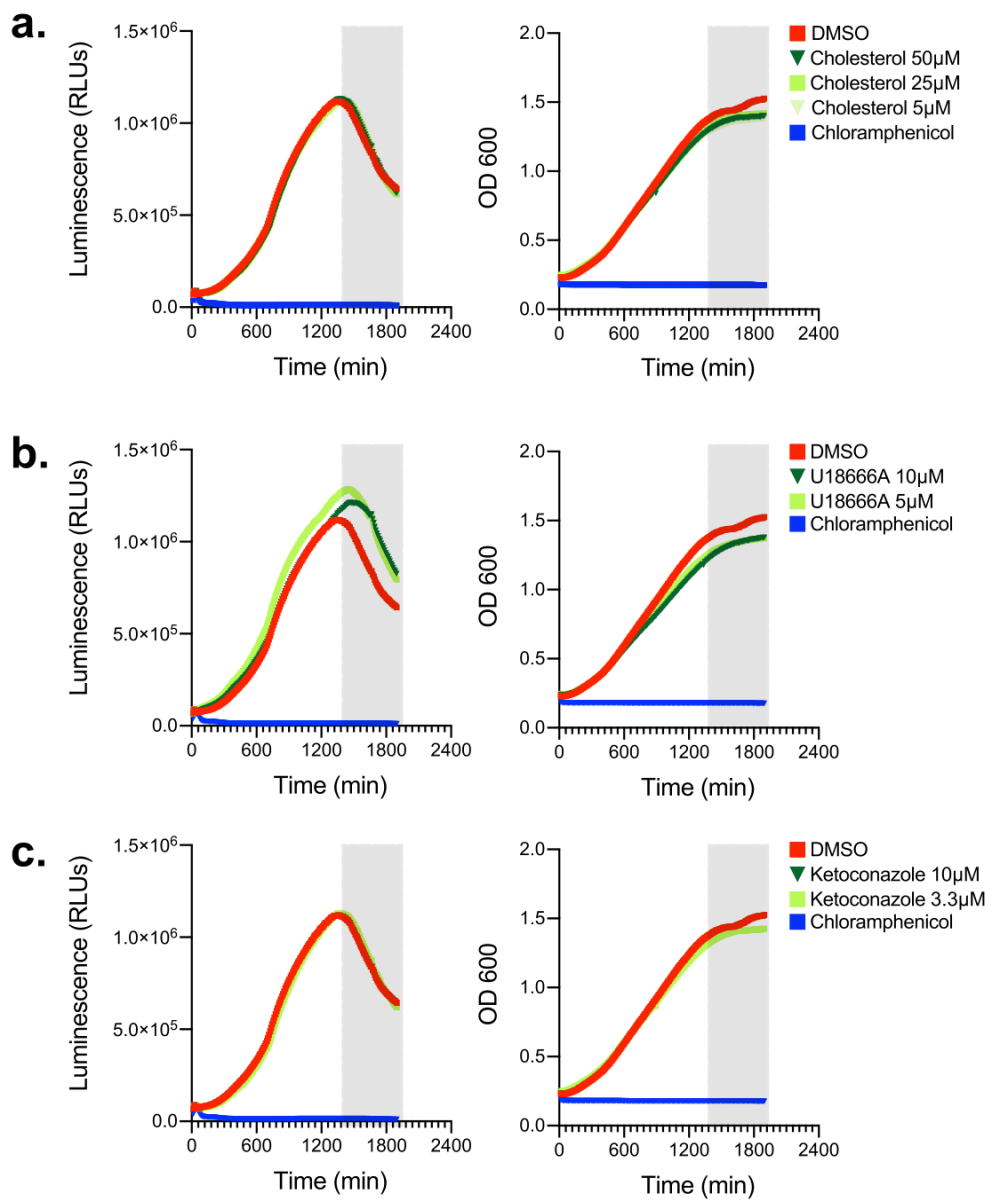
FIGURE 2: Effects of cholesterol, U18666A and ketoconazole on bacterial growth and luminescence production in axenic cultures. **(a-c)** Luminescence output of the Lp *icmRp-LuxR* measured every ten min in axenically grown AYE liquid cultures in the presence/absence of different concentration of cholesterol **(a)**, U18666A **(b)**, or ketoconazole **(c)** over the indicated time period. Each timepoint represents an average of technical triplicates. Grey panel denotes entry into stationary phase. **(a-c)** Representative data from one of three biological replicates are shown.

Because we sought to investigate how cellular sterols impact Lp intracellular replication using a pharmacological approach, we wanted to determine if the reagents we were going to use directly impact bioluminescence production or bacterial replication. First, Lp growth in AYE broth was measured in the presence of different amounts of cholesterol (from 5 to 50 µM; **Fig. 2a**) but neither bacterial replication nor bioluminescence output changed when cholesterol was added, indicating that cholesterol at the concentrations used (i) did not confer growth advantage or disadvantage for Lp and (ii) did not affect bioluminescence output (**Fig. 2a**). Next, we tested if U18666A or ketoconazole had an off-target effect in Lp that directly affects bacterial growth or bioluminescence output. U18666A inhibits the lysosomal transmembrane transporter NPC1 [53], whereas ketoconazole blocks cholesterol *de novo* biosynthesis by inhibiting demethylation of lanosterol [54]. In axenic growth cultures, U18666A treatment slightly reduced Lp replication and slightly enhanced bioluminescence output in late log phase (**Fig. 2b**), indicating that during late growth phase the bioluminescence output per bacterium increased compared to vehicle treated cells. Conversely, ketoconazole treatment affected neither bacterial replication nor bioluminescence output as the data was indistinguishable from vehicle control treated cells (**Fig. 2c**). We conclude that cholesterol and ketoconazole treatment at the concentrations used would not directly interfere with bacterial replication in the infection assay, whereas the bioluminescence output from the U18666A treatment conditions will likely overestimate slightly the number of viable bacteria.

### Perturbations in cellular cholesterol homeostasis leads to a decrease in Lp replication within BMDMs

**Figure 3:**
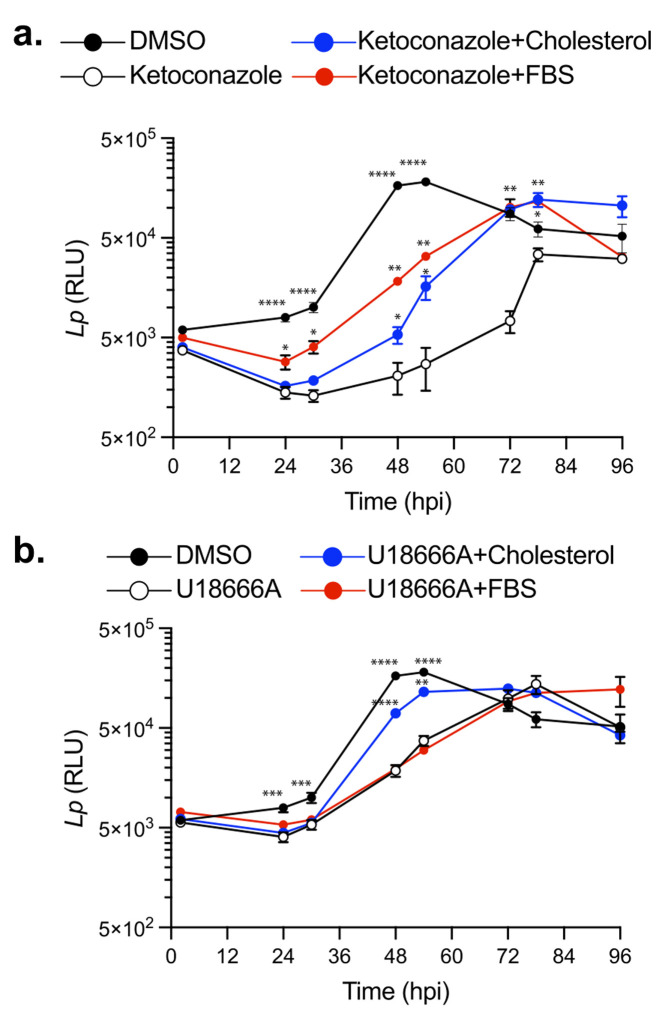
FIGURE 3: Impact of cellular cholesterol on Lp intracellular replication. Intracellular bacterial replication measured by luminescence output over 96 hrs from BMDMs infected with Lp *icmRp-LuxR* at MOI of 7.5 in the absence or presence of 3.3 µM ketoconazole **(a)**, 5µM U18666A **(b)**, 5µM cholesterol **(a-b)**, 1% (v/v) FBS **(a-b)** or DMSO **(a-b)**. Each data point represents an average of technical triplicates ± SD. For statistical analysis, each data series was compared to the data series obtained from the ketoconazole only-treated cells **(a)** or U18666a only-treated cells **(b)** using two-way ANOVA analysis. **** p<0.0001; *** p<0.0002; ** p<0.0021; * p< 0.033 **(a-b)** Representative data from one of three biological replicates are shown.

Cellular cholesterol in mammalian macrophages is primarily derived from two sources: (i) *de novo* biosynthesis at the ER and (ii) internalization of serum-derived dietary cholesterol [34]. To investigate how cholesterol homeostasis impacts Lp intracellular replication, we first disrupted cholesterol biosynthesis using ketoconazole. Because uptake of serum-derived cholesterol complements the loss of *de novo* cholesterol production in eukaryotic cells, in these experiments BMDMs were pre-cultured with serum-free (SF) RPMI overnight prior to the infection and subsequently were kept in the same media during the infection. *Legionella* intracellular growth was measured via bioluminescence output at discrete timepoints for 96 hours in the presence or absence of 3.3 µM ketoconazole, which was added at 2 hpi. In the absence of ketoconazole, bioluminescence increased ∼ 30-fold from the inoculum and the bacterial population peaked at 48 hpi (**Fig. 3a**); however, when cholesterol biosynthesis was inhibited, maximum bacterial growth was reached at 78 hpi and was reduced by ∼73% (**Fig. 3a**). These data demonstrate that loss of *de novo* cholesterol synthesis during infection significantly decreased Lp replication in macrophages suggesting that a certain amount of cellular cholesterol is critical for optimal infectivity. The defect in Lp growth under cholesterol depletion conditions was rescued when an exogenous source of cholesterol was provided by direct supplementation of either 5 µM cholesterol or serum (1% FBS v/v) in the culture media (**Fig. 3a**), indicating that cholesterol uptake by macrophages can complement the loss of cholesterol *de novo* synthesis. Because neither ketoconazole nor cholesterol altered bacterial growth in axenic cultures (**Fig. 2a** and **c**), these data are consistent with cholesterol having an effect on the host cell in a manner that impacts bacterial replication. We conclude that a certain amount of cellular cholesterol is important for optimal infectivity and loss of *de novo* cholesterol synthesis during infection decreases the capacity of macrophages to support Lp replication.

Several cellular transport mechanisms shuttle cholesterol between different membrane organelles in order to maintain organelle-specific sterol concentrations within the respective lipid bilayers. NPC1-mediated cholesterol export from lysosomes is one key step in cholesterol intracellular trafficking [55, 56]. Loss of NPC1 function results in accumulation of cholesterol within late endosomes/lysosomes and cholesterol depletion from Golgi membranes, which can alter membrane transport pathways [57]. Therefore, we investigated whether cholesterol export from lysosomes would impact Lp intracellular replication. Macrophages treated with U18666A – a NPC1 specific inhibitor [53] - exhibited a reduced capacity to support Lp replication (**Fig. 3b**), which manifested in significant delay and lower growth curve peak (**Fig. 3b**). Importantly, exogenously supplemented cholesterol that can be absorbed by cells directly via the plasma membrane partially restored the capacity of U18666A-treated macrophages to support bacterial replication (**Fig. 3b**). Conversely, serum-derived cholesterol packaged in LDLs that is extracted in lysosomes and exported by NPC1 [34] did not complemented the growth defect phenotype induced by U18666A treatment (**Fig. 3b**). These data demonstrate that cholesterol imbalance caused by a blockade in the NPC1-mediated cholesterol export pathway also decreases Lp replication in BMDMs.

To determine if cholesterol synthesis and NPC1-mediated export function in parallel to facilitate optimal bacterial replication in macrophages we treated cells simultaneously with ketoconazole (3.3 µM) and U18666A (5 µM). However, the combined treatment was poorly tolerated by macrophages under serum-free conditions in the absence of infection and resulted in significant decrease in viability which precluded the analysis of Lp intracellular growth under those conditions (data not shown). We conclude that cholesterol imbalance caused by disruption of either *de novo* synthesis or lysosomal export decreases the capacity of macrophages to support Lp intracellular growth.

### Cholesterol imbalance reduces the size of the *Legionella*-occupied intracellular niche

**Figure 4:**
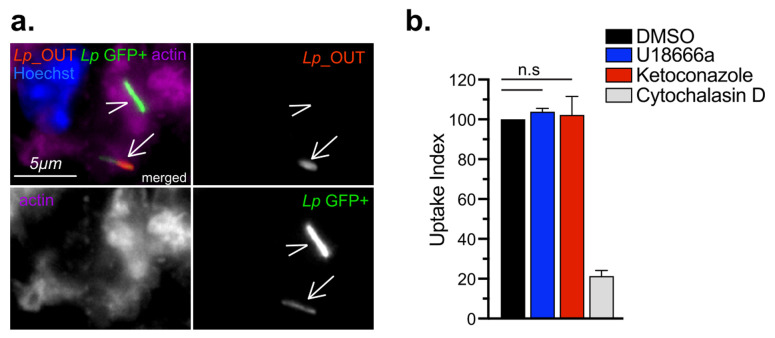
FIGURE 4: Lp uptake by U18666A- and ketoconazole-treated BMDMs. **(a)** Representative micrographs showing one completely (arrow head) and one partially internalized (arrow) bacterium. BMDMs were infected with GFP+ Lp *ΔflaA* and stained with anti-Lp antibody prior to permeabilization to distinguish intracellular (green) from surface-associated bacteria (red and green). **(b)** Phagocytosis of Lp by BMDMs pre-treated for 24 hrs with either DMSO, 3.3 µM ketoconazole or 5 µM U18666A prior to a two hr infection with GFP+ Lp *ΔflaA*. As a control, some BMDMs were treated with 5 µM cytochalasin D for the duration of the infection. Uptake index is calculated by normalizing the percentage of internalized bacteria for each condition to the percentage of internalized bacteria by the DMSO-treated cells in each experiment. Bars represent average of three biological replicates ± SD where > 100 bacteria for each treatment were scored. (n.s) not-significant; one-way ANOVA. **(a-b)** Representative data from one of two biological replicates are shown.

In cellular infections, maximal bacterial proliferation depends on a number of distinct stages of the Lp intracellular lifecycle functioning at optimal capacity, anyone of which might be impacted by imbalance in cellular cholesterol. To gain insight into the cholesterol-dependent cellular function(s) that dictate how well macrophages support *Legionella* replication, we first investigated if Lp uptake by macrophages was affected under cholesterol imbalance conditions. To this end, we treated BMDMs with ketoconazole or U18666A for 24 hrs to induce cholesterol imbalance after which Lp internalization was measured for two hpi using inside/out microscopy approach (**Fig. 4 a-b**). Neither ketoconazole nor U18666A treatment reduced the number of internalized bacteria by BMDMs, unlike the disruption of actin polymerization which interfered with phagocytosis (**Fig. 4b**). Thus, a defect in bacteria uptake is unlikely the cause for the reduced Lp growth in BMDMs infections under cholesterol imbalance conditions.

**Figure 5:**
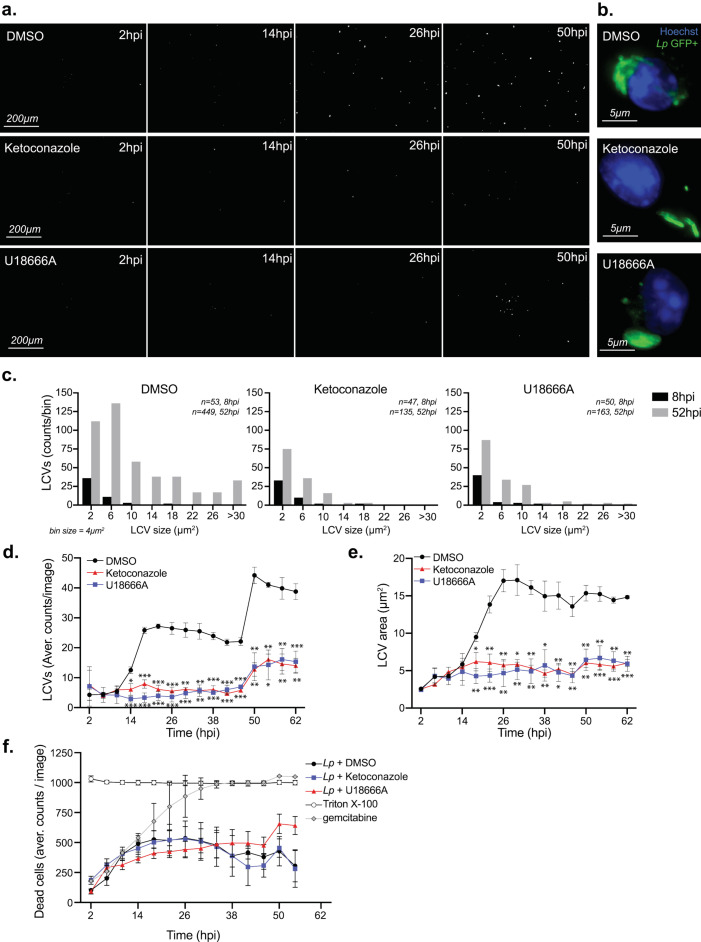
FIGURE 5: Effects of U18666A and ketoconazole on Lp uptake, dissemination and intracellular replication. **(a,c-f)** Live-cell microscopy analysis of BMDMs infected with GFP+ Lp *ΔflaA* for 62 hr. **(a)** Representative images (0.85 X 0.63mm, 0.54mm^2^) acquired at the indicated time points showing individual LCVs containing GFP-expressing Lp in a monolayer of infected BMDMs (∼1,000 cells/image) which were treated with either DMSO, 3.3 µM ketoconazole or 5 µM U18666A. **(b)** Micrographs showing representative LCVs that support bacterial replication in BMDMs infected with GFP+ Lp *ΔflaA* at twelve hpi and treated as indicated. **(c)** Distribution of LCV sizes (µm^2^) harbored by BMDMs at eight and 52 hpi. Cells were treated as indicated. The size of each bin is 4µm^2^. Cumulative data from twelve images acquired from three technical replicates for each treatment and each time-point are graphed as LCV counts/bin. **(d)** The average number of LCVs per image, **(e)** the average LCV size (µm^2^), and **(f)** the number of dead BMDMs are shown over the course of the infection. BMDMs were treated as indicated. Averages ± SD of data from twelve images from three technical replicates (four images per replicate) for each condition are shown. For statistical analysis, each data series was compared to the data series obtained from DMSO-treated cells using two-way ANOVA analysis. **** p<0.0001; *** p<0.0002; ** p<0.0021; * p< 0.033 **(f)** In control treatments, BMDMs were treated either with 0.2% Triton X-100 to determine the maximum number of dead cells or with the cytotoxic reagent gemcitabine. **(a-f)** Representative data from one of three biological replicates are shown.

Because macrophages treated with ketoconazole or U18666A internalized Lp normally, we next investigated whether cholesterol affects LCV biogenesis and expansion. To this end, we directly measured LCV dynamics in macrophage infections using live-cell microscopy by infecting BMDMs with GFP-expressing Lp and imaging of the infected cells every four hours over ∼2.5 days (**Fig. 5 a-e**). The number of LCVs as well as their respective sizes represent two parameters that were quantified for every timepoint. The distribution of LCVs by size clearly showed an increase in larger organelles as the infection progressed (eight hpi vs 52 hpi; **Fig. 5c**). The number of LCVs also increased over time and two infection cycles with progressively higher number of LCVs peaking at ∼26 and 50 hpi were observed (**Fig. 5d**). For both infection cycles, the average LCV size at the peaks were comparable (**Fig. 5e**). Together these data show detailed population-based analysis of LCV dynamics in infected BMDMs based on unbiased automated live-cell imaging.

The maturation of the LCV into a membrane-bound organelle that supports bacterial replication is completed within four hours of bacteria internalization at which point Lp starts dividing [58]. LCV maturation defects should decrease either the average LCV size or the overall number of LCVs that support bacterial replication because of a number of possible reasons – for example a delay in initiation of bacterial replication or a decrease in nutrient availability. To determine if cholesterol imbalance decreased Lp intracellular replication by causing an LCV maturation defect we directly compared the number of organelles that support Lp replication as well as their sizes under conditions of disrupted cholesterol homeostasis at an early timepoint of infection. The breakdown of LCV sizes at eight hpi - an early timepoint after initiation of bacterial replication but before bacterial egress - revealed that LCVs within ketoconazole-treated as well as U18666A-treated BMDMs were similar to the ones from vehicle-treated cells (**Fig. 5 c**), indicating that LCV maturation occurred normally despite a blockade in either *de novo* cholesterol synthesis or trafficking. At a later timepoint (52 hpi), after several rounds of infections have occurred, the number and size of the bacteria-occupied organelles was lower under cholesterol imbalance conditions (**Fig. 5c**). Significant differences in the size and number of LCVs between cholesterol imbalance conditions and vehicle control treatments were observed after 18 hpi (**Fig. 5e**). These data indicate that cholesterol imbalance lowers macrophage capacity to support Lp intracellular replication by limiting the number of bacteria each LCV produces. In principle, lower macrophage viability caused by cholesterol imbalance could potentially also restrict Lp replication by limiting the number of available bystander cells; therefore, the membrane-impermeable fluorescent dye Cytotox RED was used during the life-cell imaging experiments to identify and quantify host cell death (**Fig. 5f**). The analysis of cell viability showed similar kinetics among all treatment conditions (**Fig. 5f**), demonstrating that Lp intracellular growth defects caused by ketoconazole and U18666A treatments are unlikely a consequence of decrease in cell viability.

Taken together, these data demonstrate that inhibition of either *de novo* cholesterol biogenesis or NPC1 function interferes with LCV functionality in a manner that effectively lowers the capacity of the LCV to support optimal Lp replication resulting in fewer bacteria egressing from the host cell and participating in the subsequent infection cycle. The LCV dynamics data are also consistent with the data from the cellular infection assays that showed a growth delay rather than a growth restriction phenotype under cholesterol imbalance conditions (**Fig. 3a-b**).

### *Legionella* infection increases filipin staining in macrophages

**Figure 6:**
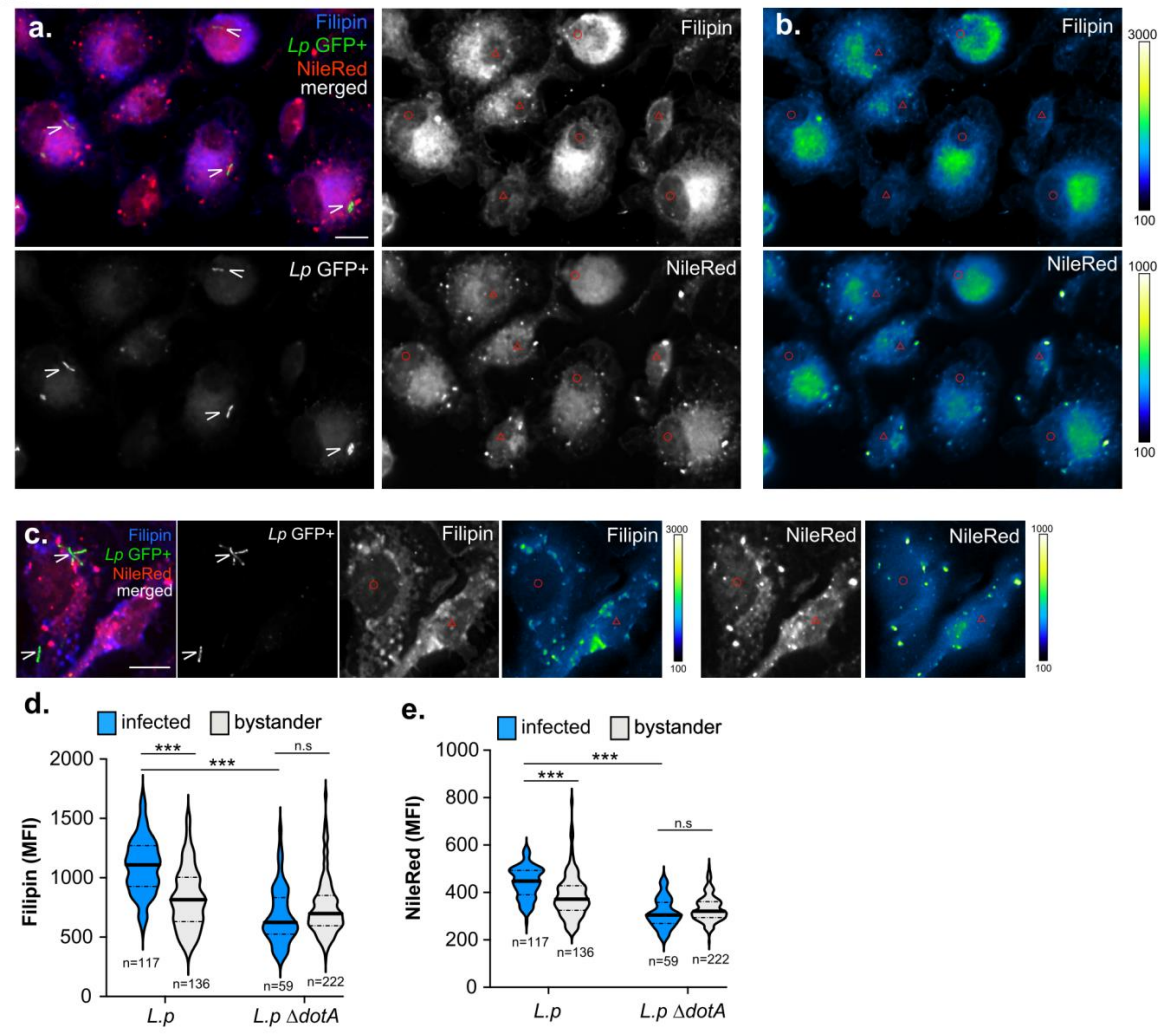
FIGURE 6: Microscopy analysis of cholesterol and neutral lipids content of BMDMs infected with *L. pneumophila*. **(a-c)** Representative micrographs of 3D projections from infected (O) and bystander (Δ) BMDMs at six hpi with GFP-expressing Lp01 Δ*flaA* (a-b) or Lp01 *ΔflaAΔdotA*. Cells were stained with Filipin and NileRed for visualization of the cellular pools of cholesterol and neural lipids, respectively. LCVs are indicated with arrow heads (>) **(b-c)** Show individual channels pseudo-colored with the Kindlmann color map. (**d-e)** Violin Plots of Filipin **(d)** and NileRed **(e)** mean fluorescent intensity (MFI) in infected and bystander macrophages at six hpi. *n*- the number of cells included in each condition. n.s – not significant, *** p<0.0001 (two-tailed unpaired T-test) **(a-e)** Representative data from one of three biological replicates are shown.

Because of the link between cholesterol imbalance and Lp intracellular replication, we sought to visualize cholesterol distribution in macrophages during infection using microscopy. To this end, we used the polyene macrolide Filipin III to visualize sterols and the lipophilic dye NileRed to image neutral lipids in macrophages. Filipin is a conventional imaging probe for cellular cholesterol that binds non-esterified cholesterol but not cholesterol esters [59]. Because filipin photobleaches easily, we compared the signal intensities of infected vs uninfected bystander macrophages which were present side-by-side and were imaged simultaneously. For these experiments, BMDMs were infected with GFP-expressing Lp strains for six hours (**Fig. 6a-e**). The sterols content among macrophages varied widely, as inferred by the filipin signal mean fluorescence intensity (MFI) in each cell (**Fig. 6d**). Nevertheless, on average filipin MFI was significantly higher in macrophages harboring bacteria by ∼25% as compared to uninfected neighboring bystander cells (**Fig. 6a-b, d**). Noticeable difference in the filipin signal distribution of infected vs bystander cells was observed where cholesterol accumulated in broad diffuse perinuclear regions, which indicates spatial regulation (**Fig. 6b**). Similar to the results with filipin, Lp-infected macrophages had ∼12% higher NileRed MFI as compared to bystander macrophages (**Fig. 6a** and **e**). NileRed is non-fluorescent in polar solvents but is intensely fluorescent in hydrophobic solvents and in cells preferentially stains neutral lipids [60, 61]. Collectively, these data indicate that sterol and neutral lipids concentrations are higher in *Lp*-infected BMDMs as compared to bystander uninfected macrophages.

Because Lp has been shown to stimulate pro-lipogenic pathways in infected BMDMs in a T4SS-dependent manner [29, 32], we investigated whether the *L.p* Dot/Icm apparatus is required for the elevated lipid concentration in infected cell. To this end, BMDMs were infected with a clean deletion mutant missing the *dotA* gene, whose gene product is an essential component of the Dot/Icm apparatus and therefore lacks a functional T4SS [62]. The average MFIs of BMDMs harboring L.p Δ*dotA* bacteria for both filipin and NileRed were indistinguishable from the average MFIs of uninfected bystander cells (**Fig. 6c-e**). We conclude that the Dot/Icm apparatus is required for the elevated lipid concentration in infected cell. The Dot/Icm dependency of the phenotype indicates that Lp might directly or indirectly modulate one of more host processes that increase non-esterified sterols and neutral lipids in infected cells.

### Exogenous cholesterol potentiates *Legionella* intracellular replication in macrophages

**Figure 7:**
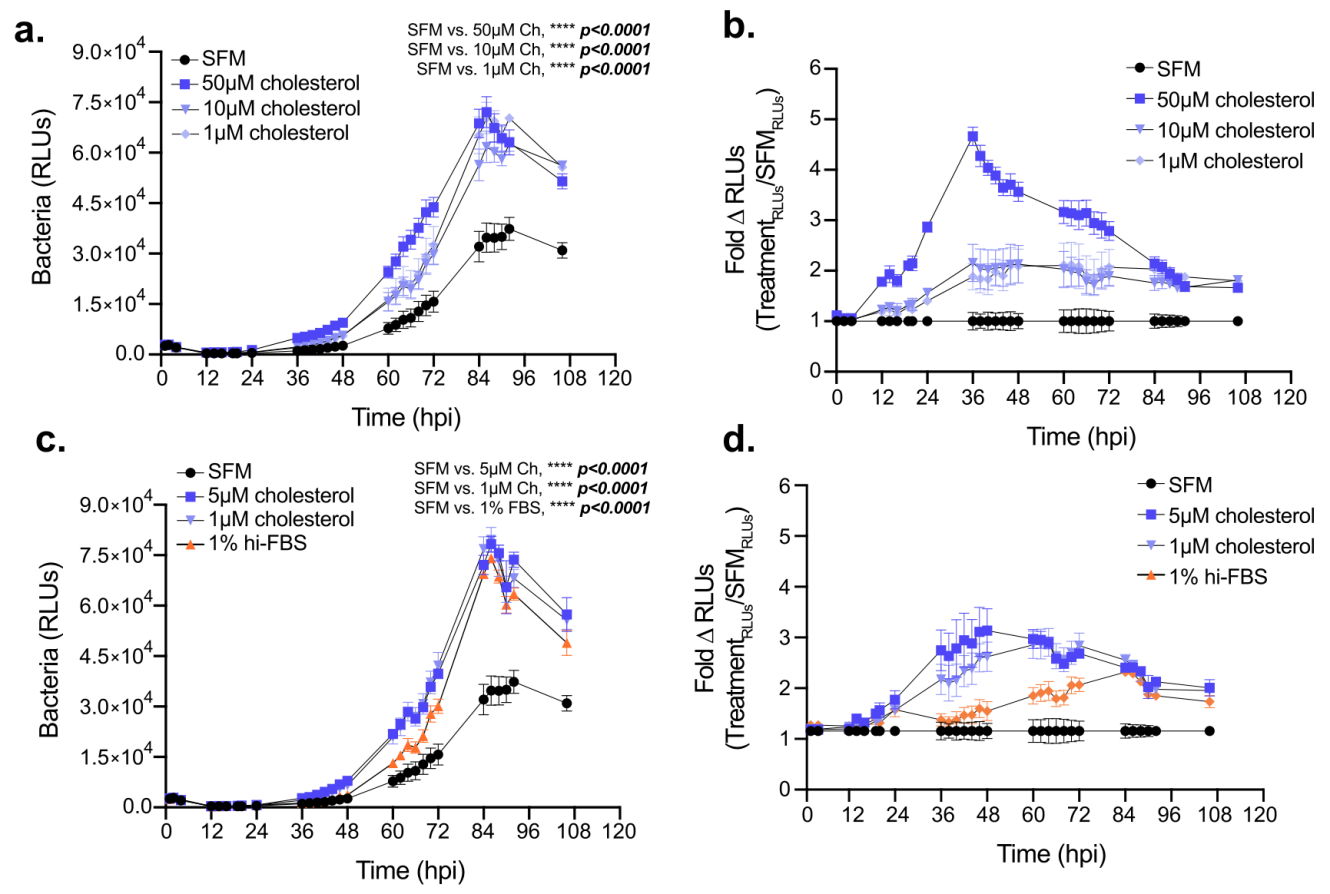
FIGURE 7: Effects of cholesterol supplementation on Lp intracellular replication. Intracellular bacterial replication measured by bioluminescence output over 108 hrs from BMDMs infected with Lp *icmRp-LuxR* (MOI of 2.5). Cells were either cultured with the indicated amounts of cholesterol for 24 hrs prior to infection **(a-b)** and infected in serum-free media (SFM) or were treated as indicated for the duration of infection starting at 2hpi **(c-d)**. **(a** and **c)** Luminescence output from infected BMDMs measured at the indicated times post infection. **(b** and **d)** Ratiometric analysis of Lp intracellular replication in the presence/absence of cholesterol or FBS. For each time point the ratio represents the bacteria-derived bioluminescence output produced when BMDMs were cultured under the indicated treatments divided by the output produced when BMDMs were cultured in SFM. **(a-d)** shown are averages from technical triplicates ± SD for each time-point. **** p<0.0001, two-way ANOVA. **(a-d)** Representative data from one of three biological replicates are shown.

Elevated cellular cholesterol in Lp infected cells coupled with the sub-optimal intracellular bacterial replication under cholesterol-limiting conditions raised the question of whether higher cellular cholesterol concentration benefits bacterial growth in macrophages. Thus, we investigated Lp intracellular growth in BMDMs under high cholesterol conditions by culturing the macrophages with various amounts of exogenously supplied cholesterol for 24 hrs prior to infection while the infection was carried out without cholesterol supplementation (**Fig. 7a-b**). Pre-loading BMDMs with cholesterol was sufficient to increase their capacity to support Lp growth over several days in a dose-dependent manner (**Fig. 7a, b**). Similarly, enhanced bacterial replication was observed when cholesterol was supplemented at the time of infection (**Fig. 7c, d**). Because cholesterol itself did not alter Lp replication in axenic cultures (**Fig. 2a**), these data indicate that the growth-promoting effects of cholesterol on Lp intracellular replication likely occur as a result of alterations in the host cell. Thus, we conclude that increasing cellular cholesterol enhances the capacity of BMDMs to support *Legionella* replication.

## DISCUSSION

Our work here identified a link between Lp intracellular growth and cholesterol homeostasis in the host cell by demonstrating that cholesterol imbalance induced through disruption of *de novo* production or trafficking in infected macrophages reduced capacity of macrophages to support Lp growth by decreasing the number of bacteria produced from each LCV. Several lines of evidence are consistent with a decreased LCV housing capacity under cholesterol imbalance as the cause for this phenotype: (1) treatment with either ketoconazole or U18666A reduced Lp replication in macrophage infection assays, while Lp uptake and LCV maturation were not affected by cholesterol imbalance; (2) exogenous cholesterol complemented the Lp growth defect induced by ketoconazole or U18666A treatments; (3) macrophages pre-loaded with exogenous cholesterol supported Lp growth better; (4) macrophages under cholesterol imbalance produced on average less bacteria within LCVs over the course of several infection cycles; (5) after the primary infection cycle, less LCVs were observed under cholesterol imbalance conditions in the subsequent infection cycles. Thus, we propose that one or more cellular processes that determine optimal Lp replication within the LCV is regulated directly or indirectly by cholesterol.

The intracellular replication of several vacuolar bacterial pathogens, such as *C. burnetii* and *Chlamydia trachomatis*, have been shown to be impacted either positively or negatively by imbalance in cellular cholesterol [39]. For instance, U18666A treatment reduced *C. trachomatis* intracellular replication in HEP-2 cells by interfering with cholesterol delivery through CD63-positive multivesicular bodies to the *Chlamydia*-containing vacuole [63]. *C. trachomatis* harvests cholesterol from the host cell and incorporates it in the bacterial cell envelope and thus depends on cholesterol for optimal growth [42]. It is unlikely that Lp acquires cholesterol as a nutrient or for macromolecular biosynthesis because it is evident from our data that Lp growth rate in axenic cultures did not increase upon cholesterol supplementation. Indeed, it is well established that Lp preferentially catabolizes amino acids and carbohydrates for macromolecular biosynthesis [64–66]. U18666A treatment accumulates cholesterol in the late endosomal/lysosomal compartment increasing the sterol content of *C. burnetii*-occupied vacuoles, which continuously intercept endocytic and lysosomal vesicular traffic [43, 67, 68]. In this case, cholesterol was shown to be bacteriolytic [67]. We did not detect obvious accumulation of filipin on Lp or in the direct proximity of the LCV in infected macrophages as it has been observed for *Coxiella* [43]and *Chlamydia* [42], indicating that cholesterol is unlikely to have been incorporated in the Lp cell envelope or increased drastically within the LCV membrane. Therefore, we speculate that the cholesterol-dependent processes which promote Lp replication might occur at an organelle membrane distinct from the LCV.

The differences in biogenesis and homeostasis mechanisms that govern the intracellular niches of different bacterial pathogens can explain the distinct cholesterol-dependent phenotypes observed during infection. Both *C. trachomatis* and *C. burnetii* reside within vacuoles that intercept trafficking from or are derived from cellular organelles with high cholesterol content (Golgi apparatus and lysosomes, respectively), whereas LCVs fuse with the ER, a low cholesterol content organelle. Cholesterol produced at the ER is tightly regulated within a narrow range and a majority of the lipid is rapidly exported through vesicular and non-vesicular transport mechanisms resulting in significantly lower cholesterol ER membrane content relative to other organelles [34, 69]. Minute changes in cholesterol at the ER trigger homeostasis mechanism that trap excess cholesterol within lipid droplets or initiate *de novo* biogenesis under lipid starvation conditions [34]. Lp infected macrophages had a distinct broadly defused perinuclear increase in filipin signal as compared with neighboring bystander cells suggesting that Lp may subvert directly or indirectly cellular processes that control cholesterol homeostasis. Indeed, Lp T4SS effectors have been shown to activate the pro-lipogenic MTOR signaling cascade [29, 32]. Interference with MTOR function decreases the LCV housing capacity and results in premature LCV rupture [29]. Therefore, the defects in LCV homeostasis induced by cholesterol imbalance and MTOR inhibition overlap to a certain extend. Interestingly, the LCV instability caused by MTOR blockade was complemented by fetal bovine serum (FBS) containing LDLs but not by FBS depleted of cholesterol [29]. Perhaps, cholesterol is a positive regulator of LCV expansion, which would explain the normal Lp uptake and LCV maturation under cholesterol imbalance as well as the higher Lp intracellular growth observed in the presence of exogenous cholesterol.

How does cholesterol imbalance decrease LCV housing capacity? Cholesterol regulates protein functions by several mechanisms including membrane recruitment of cholesterol-binding proteins - such as Syntaxin 6 (Stx6) and Caveolin - as well as higher-order clustering and assembly of protein complexes within lipid-ordered microdomains in lipid membranes [33]. Large number of Lp T4SS effectors localize to host organelle membranes [70–72]. For example, SidC and DrrA/SidM contain PtdIns(4)P-binding domains, which dictate recruitment to the PtdIns(4)-P enriched LCV membrane [73, 74], other effectors achieve membrane localization through lipidation [71]. The presence of cholesterol/ergosterol binding domains given the large Dot/Icm effector repertoire is a possibility, though none have been discovered yet. Nearly 25% of Lp T4SS effectors encode one or more transmembrane domains [75]; thus, disruption of cholesterol-dependent lipid microdomains at the LCV or at other cellular organelles might interfere with effector functions by altering membrane fluidity. For example, iron transport through the eight transmembrane domain effector MavN, which is essential for Lp intracellular replication and resides within a cholesterol-containing region of the LCV membrane [76] might be affected. Regulation of Lp growth by cholesterol strictly via host specific factors is also a possibility. The cholesterol-binding Q-SNARE Stx6 redistributes from the trans-Golgi network to a Rab11+ recycling endosomes upon cholesterol accumulation in the endosomal compartment brought by a blockade in NCP1-mediated cholesterol export resulting in integrins recycling and cell migration defects [57]. Similarly, cholesterol imbalance might alter trafficking pathways critical for LCV expansion.

Although cellular cholesterol maximizes Lp growth within LCVs, overabundance of cholesterol can rigidify biological membranes causing cellular stress and cytotoxicity unless cholesterol is acylated, extracted and packaged within lipid droplets [77]. Interestingly, Lp secretes three GDSL-type esterase/lipases (PlaA, PlaC and PlaD) that have been shown to acylate ergosterol as well as cholesterol *in vitro* [26]. These enzymes are secreted by the T2SS either in the extracellular milieu or in the lumen of the LCV after bacteria are internalized and therefore are likely to access and presumably modulate cholesterol within the plasma membrane as well as the LCV membrane [28]. Cholesterol esterification in infected cells by the Lp PlaA/C/D enzymes has not been demonstrated, however PlaA can destabilize the LCV membrane when the T4SS effector SdhA is deleted [23], indicating that overt cholesterol extraction from the LCV membrane after cholesterol acylation by PlaA could be a destabilizing event. The precise mechanism(s) by which cholesterol imbalance regulates LCV housing capacity clearly warrants further investigation.

## MATERIALS AND METHODS

### Bacterial Strains

All strain used in this study were derived from the *Legionella pneumophila* serogroup 1, strain Lp01 [19]. To avoid NLRC4-mediated pyroptosis triggered by flagellin when BMDMs from C57BL/6J mice are infected by *Legionella* that express flagellin, strains used in this study have a clean deletion of the *flaA* gene. The following strains on the *L.* pneumophila Lp01 background were used in this study: (1) Isogenic clean deletions strain *Lp01* Δ*flaA* was produced by allelic exchange (in this study); (2) Isogenic clean deletions strain *Lp01* Δ*dotA* p*Tac*::GFP expressing GFP under isopropyl-beta-D-thiogalactoside (IPTG)-inducible promoter; (3) Isogenic clean deletions strain *Lp01* Δ*flaA* p*Tac*::GFP expressing GFP under IPTG-inducible promoter; (4) *Lp01* Δ*flaA-LuxR* strain, referred to as Lp *icmRp-LuxR* in which the LuxR operon (*luxCDABE)* from *Photorhabdus luminescens* was inserted via homologous recombination on the bacterial chromosome downstream of the *icmR* promoter (in this study).

*Legionella* strains were grown for two days at 37^°^C on charcoal yeast extract (CYE) plates (1% yeast extract, 1%*N*-(2-acetamido)-2-aminoethanesulphonic acid (ACES; pH 6.9), 3.3 mM l-cysteine, 0.33 mM Fe(NO3)_3_, 1.5% bacto-agar, 0.2% activated charcoal) [78]. For all infections, *Legionella* were harvested from CYE plates and grown in liquid cultures. For liquid cultures, bacteria from day two heavy patches grown on CYE plates were suspended to optical densities of 0.1 or 0.3 in 2.5 mL of complete ACES buffered yeast extract (AYE) broth (10 mg/mL ACES: pH 6.9, 10 mg/mL yeast extract, 400 mg/L L-cysteine, 135 mg/L) supplemented with 100 µg/mL streptomycin and grown aerobically at 37°C to early stationary phase (between 18-22 hours, to OD 3.0-4.0). Liquid cultures of the GFP-expressing strains were supplemented with 10 μg/mL chloramphenicol.

### Plasmids and strain construction

The GFP-expressing bacterial strains were generated by electroporation with the pAM239 plasmid, which carries chloramphenicol resistance and encodes GFP under the IPTG-inducible Ptac promoter. Transformants were selected on CYE chloramphenicol plates and GFP expression was verified by microscopy.

The pSR47::P_*icmR*_-LuxR plasmid was generated by cloning 875 nt sequence upstream of the *icmR* gene (P_*icmR*_) and the *luxCDABE* (LuxR) operon into the pSR47 suicide plasmid. The 875 nt P_*icmR*_ genomic sequence was amplified with a forward cloning primer containing an EcoRI restriction enzyme site (5′-CGGAATTCGTCCGGGGTATTAACACTTAGG-3′) and a reverse cloning primer containing a BamHI restriction enzyme site (5′-CGGGATCCTATTACCACTCCTGAGCTAAATCTC-3′). The LuxR operon was excised from pXen-13 (Xenogen) by double digest with BamHI and NotI restriction enzymes. The pSR47::P_*icmR*_-LuxR plasmid was assembled in a three-way ligation reaction with pSR47 (digested with EcoRI/NotI), P_*icmR*_ (digested with EcoRI/BamHI) and LuxR (digested with BamHI/NotI). To integrate the LuxR operon in the *Legionella* chromosome, the pSR47::P_*icmR*_-LuxR was introduced in Lp via tri-parental mating and clones that have undergone homologous recombination were selected on CYE plates containing kanamycin and streptomycin and tested for bioluminescence.

The Δ*flaA* allele was generated by ligation of ∼1kb regions upstream and downstream of *lpg1340* (*flaA/FliC*). To this end, the upstream region flanked by BamHI and XhoI restriction sites was PCR amplified using Lp genomic DNA as a template and the forward primer (5′-CGGGATCCTTCGTTGAAAGCCTTCTGGC-3′) and the reverse primer (5′-CCGCTCGAGTCTCCTCAGACCTGAATCC-3′). Similarly, the downstream region flanked by XhoI and NotI restriction sites was produced using the forward primer (5′-CCGCTCGAGGGATGTCGCAATCGAAGTGC-3′) and the reverse primer (5′-AATGCGGCCGCAGTTAATGAATTCACTCCC-3′). Both fragments were digested with the respective restriction enzymes and were ligated in the gene replacement vector pSR47s, which was digested with BamHI and NotI to create pSR47s-CD1340. FlaA deletion strains were generated by allelic exchange of *flaA* with Δ*flaA* after the pSR47s-CD1340 was introduced in Lp via tri-parental mating, as previously described [79].

### Reagents

The following reagents were purchased from Cayman Chemicals - u18666A (cat #10009085), ketoconazole (cat #15212), Filipin III (cat #70440), NileRed (cat # 30787). Gemcitabine (cat #G6423) and cholesterol (cat #C8667-1G) were purchased from Sigma.

### Mice

C57BL/6J mice were purchased from Jackson Laboratories and housed at LSUH-Shreveport animal facility. Ethical approval for animal procedures for experiments in this study was granted by the Institutional Animal Care and Use Committee at LSUHSC-Shreveport (protocol# P-15-026).

### BMDMs derivation and culture

Bone marrow progenitors isolated from C57BL/6J mice were cultured on 10 cm petri dishes in RPMI 1640 with L-glutamine (BI Biologics, cat #01-100-1A) supplemented with FBS (10% final volume; Atlas Biologics, cat #FS-0500-AD), conditioned medium from L929 fibroblast cells (ATCC, CCL-1; 20% final volume) and penicillin/streptomycin (VWR, cat #K952) at 37°C with 5% CO_2_. Additional media was added at days three, five and seven from the start of differentiation. Macrophages were collected and seeded for infections at day nine.

### Microscopy analysis of cellular cholesterol and neutral lipids in BMDM infections

For infections, BMDMs were seed on cover slips in re-plating media (RPMI 1640 with L-glutamine, 10% FBS, 10% M-CSF-conditioned media) for two hrs at 2.5×10^5^ cells/well seeding density in 24-well plates. Next, cells were cultured in serum-free RPMI (SF-RPMI) for 14 hrs prior to infection. BMDMs were infected with liquid culture grown GFP-expressing *Lp01* Δ*dotA* or *Lp01* Δ*flaA* bacteria for six hours at MOI of ten. At 60 min post infection, extracellular bacteria were removed by washing 5X with warm (37°C) phosphate-buffered saline (PBS) and the infected BMDMs were cultured in in SF-RPMI supplemented with 2 mM IPTG. At the end, cells were washed with warm PBS (3X) and fixed with 2% paraformaldehyde for 20 min.

For quantitative analysis of cellular cholesterol and neutral lipids, cells were stained with Filipin III (0.5 mg/mL) and 200 nM NileRed in PBS for 16 hrs at 4°C. Coverslips were subsequently washed with PBS (5X) and mounted with ProLong Gold antifade reagent (ThermoFisher) onto glass slides. Images were captured with inverted wide-field Nikon Eclipse Ti microscope controlled by NES Elements v4.3 imaging software (Nikon) using a 60X/1.40 oil objective (Nikon Plan Apo λ), LED illumination (Lumencor) and CoolSNAP MYO CCD camera. Image acquisition parameters - Filipin III (ExW 395 / EmW 455); GFP (ExW 470 / EmW 525); NileRed (ExW 555 / EmW 605). The z-axis acquisition was set based on the out-of-focus boundaries and the distance between individual Z-slices was kept at 0.3 µm. Image analysis was performed with NES Elements v4.3 imaging software. Only linear image corrections in brightness or contrast were completed. For all analyses, three-dimensional images of randomly selected fields were acquired where image acquisition parameters were identical for all cover slips from the same experiment. A binary cell outline mask was created based on NileRed fluorescence from z-axis image projection and the MFI of Filipin III and NileRed for each cell was calculated. Background MFI for each channel was individually determined for each acquired field from a cell-size mask positioned in an unoccupied area, which was subsequently subtracted from the MFI of each cell in the field.

### Macrophage uptake assay

BMDMs were seeded on cover slips as described in the previous section. Following attachment, cells were cultured in SF-RPMI containing either 3.3 µM ketoconazole or 5 µM U18666A or vehicle volume equivalent (DMSO) for 24 hrs. Next, BMDMs were infected with liquid culture grown *Lp01* Δ*flaA* p*Tac*::GFP at MOI = 10 for two hrs in the presence/absence of the inhibitors. One set of cells were treated with 5 µM cytochalasin D at 30 min prior to the infection to block phagocytosis.

After cells were washed with warm PBS (3X), the infection was stopped by the addition of 2% paraformaldehyde for 60 min at ambient temperature. The surface-associated bacteria were immunolabeled prior to plasma membrane permeabilization with chicken α-*Legionella* IgY antibody [29] for 90 min in goat serum containing PBS (2% vol/vol). Next, cover slips were washed with PBS (3X), fixed with 2% PFA for 60 min at ambient temperature and cells were permeabilized with 0.1% Triton X-100 for 20 min. Samples were stained with tetramethyl rhodamine conjugated goat α-chicken IgY (ThermoFisher, cat# A16059) at 1:500 dilution and the high affinity actin probe Phalloidin-iFluor 633 (Cayman Chemicals, cat# 20554) at 1:3000 dilution for 60 min in goat serum containing PBS (2% vol/vol). Coverslips were mounted with ProLong Glass Antifade Mountant (ThermoFisher, cat# P36984) onto slides.

Microscopy analyses of infected cells. Images were acquired with inverted wide-field microscope (Nikon Eclipse Ti) controlled by NES Elements v4.3 imaging software (Nikon) using a 60X/1.40 oil objective (Nikon Plan Apo λ), LED illumination (Lumencor) and CoolSNAP MYO CCD camera. Image acquisition and analysis was completed with NES Elements v4.3 imaging software. For all analyses, three-dimensional images of randomly selected fields were acquired, and image acquisition parameters were kept constant for all cover slips from the same experiment. The Z depth acquisition was set based on the out-of-focus boundaries and the distance between individual Z-slices was kept at 0.3 µm. Only linear image corrections in brightness or contrast were completed. For each condition, over 100 bacteria were imaged and scored as either intracellular (single positive – green only) or not-internalized (double positive – green/red). The uptake index for each condition was calculated by dividing the percentage of intracellular bacteria from inhibitor treated cells by the percentage of intracellular bacteria from the vehicle treated cells and the result was multiplied by 100.

### *Legionella* axenic growth assays

Liquid cultures of *Lp01 icmRp-LuxR* in complete AYE were set-up at starting OD_600_ of 0.4 from plate grown bacteria (day two heavy patches) and were distributed in white-wall clear-bottom 96-well plates (Corning, cat# 3610). All conditions in all assays were performed in technical triplicates. Plates were incubated in a luminometer (Tecan Spark) at 37°C for 24 hrs. Luminescence and optical density (OD at 600 nm) data were automatically collected every five mins after the cultures were agitated for 180 sec (double orbital rotation, 108 rpms). Bioluminescence output from each well was acquired for one sec and presented as total relative light unit (RLU) counts/s.

### Assays for *Legionella* intracellular replication

For bioluminescence-based intracellular growth assays, BMDMs were seeded at 1.0×10^5^ cells per well in white-wall clear-bottom 96-well plates (Corning cat# 3610) in re-plating media (RPMI 1640 with L-glutamine, 10% FBS, and 10% M-CSF-conditioned media) for two hours. Next, cells were serum starved for 18 hours prior to infection with serum-free RPMI 1640 with L-glutamine (SF-RPMI). The cells were then infected with liquid culture grown *Lp01 icmRp-LuxR* as stated in the figure legends. Various inhibitors and/or cholesterol were added at two hpi in SF-RPMI. In cholesterol pre-loading experiments, BMDMs were seeded for infection (as detailed above) and treated with cholesterol-containing SF-RPMI for 14 hrs followed by incubation with SF-RPMI for 14 hrs prior to infection. Infections of cholesterol pre-loaded cells were carried out in SF-RPMI. Plates were kept in a tissue culture incubator at 37°C and 5% CO_2_ and periodically the bioluminescence output from each well was acquired (integration time of five sec) and presented as total RLU counts per well (Tecan Spark plate reader).

For colony forming units (CFUs) growth assay, infections were carried out in 48-well plates using three technical replicates for each condition. BMDMs were seeded at 2.0×10^5^ cells per well in re-plating media (RPMI 1640 with L-glutamine, 10% FBS, and 10% M-CSF-conditioned media) for two hours. Next, cells were cultured in RPMI 1640 with L-glutamine and 10% FBS. BMDMs were infected at MOI of two with liquid culture grown bacteria. The plate was centrifuged for five min at 1,000 rpm to bring the bacteria in contact with the macrophages. At discrete timepoints, the media from each well was collected, macrophages were lysed by adding back sterile water for ten min, and the total contents of the well (media + water) were serially diluted and plated on CYE plates. The recovered Lp CFUs were enumerated for each condition. For each timepoint, the data is represented as fold change of CFUs over the CFUs recovered from the inoculum.

### IncuCyte™ S3 automated microscopy analysis of *Legionella* intracellular replication

For infections, BMDMs were seeded in 96-well black-wall clear-bottom plates (Corning cat# 3904) at 8.0×10^4^ cells per well in re-plating media (RPMI-1640 with L-glutamine, 10% FBS, and 10% M-CSF-conditioned media) for two hours and then were serum starved for 14 hrs in serum-free phenol red-free DMEM (Gen Clone 25-501C; SF-DMEM). All infections were carried out in SF-DMEM supplemented with 2 mM IPTG and IncuCyte Cytotox™ live/dead cell RED reagent (according to the manufacturer's directions). Inhibitors were added at the time of infection. Final media volume was 150 µL per well. In each experiment, all conditions were performed in technical triplicates. Plates were centrifuged (1,000 rpms, five min) and were loaded into the IncuCyte S3 housing module. The IncuCyte™ S3 HD live-cell imaging platform (Sartorius) is a wide-filed microscope mounted inside a tissue culture incubator and is run by the IncuCyte control software. For each well, four single plane images in bright field, green (ExW 440-480nm/EmW 504-544nm) and red (ExW 565-605nm/EmW 625-705nm) channels were automatically acquired with *S* Plan Fluor 20X/0.45 objective every four hours. Image were analyzed with the IncuCyte Analysis software. For LCV analysis, bacterial fluorescence was used to generate a binary mask to define individual LCV objects and to measure the object's area size (µm^2^) and the number of objects per image. For analysis of BMM viability, accumulation of the DNA-binding fluorescent Cytotox™ RED reagent in the nucleus was used to identify dead cells. Cytotox™ RED reagent is membrane impermeable and becomes fluorescent when it binds DNA. The Incucyte S3 imaging analysis was performed in the Innovative North Louisiana Experimental Therapeutics program (INLET) core facility at LSU Health – Shreveport.

### Statistical analysis

Calculations for statistical differences were completed with Prism v9 (GraphPad Software).

## Abbreviations:

BMDM: – bone marrow-derived macrophage,
ER: – endoplasmic reticulum,
hpi: – hours post infection,
LCV: – Legionella-containing vacuole,
LDL: – low-density lipoprotein,
Lp: – Legionella pneumophila,
MFI: – mean fluorescens intensity,
MOI: – multiplicity of infection,
MTOR: – mechanistic target of rapamycin,
T2SS: – type II secretion system.
